# Computer Skills and Internet Use in Adults Aged 50-74 Years: Influence of Hearing Difficulties

**DOI:** 10.2196/jmir.2036

**Published:** 2012-08-24

**Authors:** Helen Henshaw, Daniel P A Clark, Sujin Kang, Melanie A Ferguson

**Affiliations:** ^1^NIHR National Biomedical Research Unit in HearingSchool of Clinical SciencesUniversity of NottinghamNottinghamUnited Kingdom; ^2^NIHR National Biomedical Research Unit in HearingNottingham University Hospitals NHS TrustNottinghamUnited Kingdom

**Keywords:** hearing loss, hearing difficulties, screening, intervention, hearing health care, eHealth, personal computer, Internet use.

## Abstract

**Background:**

The use of personal computers (PCs) and the Internet to provide health care information and interventions has increased substantially over the past decade. Yet the effectiveness of such an approach is highly dependent upon whether the target population has both access and the skill set required to use this technology. This is particularly relevant in the delivery of hearing health care because most people with hearing loss are over 50 years (average age for initial hearing aid fitting is 74 years). Although PC skill and Internet use by demographic factors have been examined previously, data do not currently exist that examine the effects of hearing difficulties on PC skill or Internet use in older adults.

**Objective:**

To explore the effect that hearing difficulty has on PC skill and Internet use in an opportunistic sample of adults aged 50-74 years.

**Methods:**

Postal questionnaires about hearing difficulty, PC skill, and Internet use (n=3629) were distributed to adults aged 50-74 years through three family physician practices in Nottingham, United Kingdom. A subsample of 84 respondents completed a second detailed questionnaire on confidence in using a keyboard, mouse, and track pad. Summed scores were termed the “PC confidence index.” The PC confidence index was used to verify the PC skill categories in the postal questionnaire (ie, never used a computer, beginner, and competent).

**Results:**

The postal questionnaire response rate was 36.78% (1298/3529) and 95.15% (1235/1298) of these contained complete information. There was a significant between-category difference for PC skill by PC confidence index (*P*<.001), thus verifying the three-category PC skill scale. PC and Internet use was greater in the younger respondents (50-62 years) than in the older respondents (63-74 years). The younger group’s PC and Internet use was 81.0% and 60.9%, respectively; the older group’s PC and Internet use was 54.0% and 29.8%, respectively. Those with slight hearing difficulties in the older group had significantly greater odds of PC use compared to those with no hearing difficulties (odds ratio [OR]=1.57, 95% confidence interval [CI] 1.06-2.30, *P*=.02). Those with moderate+ hearing difficulties had lower odds of PC use compared with those with no hearing difficulties, both overall (OR=0.58, 95% CI 0.39-0.87, *P*=.008) and in the younger group (OR=0.49, 95% CI 0.26-0.86, *P*=.008). Similar results were demonstrated for Internet use by age group (older: OR=1.57, 95% CI 0.99-2.47, *P*=.05; younger: OR=0.32, 95% CI 0.16-0.62, *P*=.001).

**Conclusions:**

Hearing health care is of particular relevance to older adults because of the prevalence of age-related hearing loss. Our data show that older adults experiencing slight hearing difficulty have increased odds of greater PC skill and Internet use than those reporting no difficulty. These findings suggest that PC and Internet delivery of hearing screening, information, and intervention is feasible for people between 50-74 years who have hearing loss, but who would not typically present to an audiologist.

## Introduction

The use of personal computers (PCs) and the Internet to provide health care and health-related information to patients and the public has increased substantially over the last decade [1,2], to the point where the Internet is now a major source of health information [2]. Hearing health is no exception. Current examples of PC and Internet delivery of hearing health care include hearing screening [3-5], auditory training [6], counseling [7], education [8], and information delivery [9,10]. A review of eHealth in audiology suggests that published evidence assessing online hearing health care finds these applications to be both reliable and effective [11].

Sensorineural hearing loss (SNHL) is highly associated with age, sex, and socioeconomic status (SES) [12]. Hearing loss affects approximately 1 in 4 people (27%) between 55-74 years, it increases in severity with age, is worse in males than females, and is more prevalent in those with lower SES [13]. This is a growing problem in our aging society, with the number of people with SNHL set to increase even further in the future [12,14]. The main clinical management intervention for people with hearing loss is hearing aids, but most adults aged 55-74 years (80%) do not use them [12]. The reasons for this are broad and varied, including inadequate access to audiology services because of low referral rates by family practice physicians or general practitioners (GPs), a perception that poor hearing is not that bad, acceptance of hearing loss as a normal consequence of aging, a lack of awareness of hearing loss and what to do about it, and simply not wanting to address the issue [12]. The average age of a first-time hearing aid user is 74 years, yet these adults may have suffered with significant hearing loss for an average of 10 years before receiving hearing aids [12]. A major advantage of delivering hearing health care through the Internet is the potential to increase accessibility to large numbers of people with hearing loss, many of whom do not, or cannot, access current hearing health care sources.

Hearing aids are not the only form of intervention available for hearing loss. Computerized auditory training, such as Listening and Communication Enhancement (LACE) [15], which can change a person’s ability to process sounds and can improve auditory performance on a trained task [16], is an example of an alternative intervention strategy that may help alleviate hearing difficulties [17,18] and is ideally suited for Internet delivery [6]. Other types of online hearing-related interventions include Internet discussion groups, counseling, and information provision. These interventions have demonstrated effectiveness in improving self-reported hearing abilities and satisfaction with amplification in experienced hearing aid users (mean age 63.5 years) [8], and in reinforcing positive adjustment behaviors in new hearing aid users (mean age 68 years) [7]. Yet online hearing information and hearing health care can only be effective if they are accessible and usable by the target population. The earlier an adult with SNHL begins a program of rehabilitation, the greater chance their hearing disability will be reduced and their quality of life improved [19]. Currently, there are no guidelines for the screening of age-related hearing loss for adults despite the success of newborn and school-based hearing screening programs that have been highly effective in identifying children who are deaf or hearing impaired [20,21]. Online hearing screening for adults (eg, the UK Action on Hearing Loss hearing screen [3] and the Dutch functional hearing-screening test [5]) at the onset of hearing difficulties (typically around 50 years of age) may offer a cost-effective strategy to promote early diagnosis of progressive SNHL and, in turn, may lead to earlier intervention and better quality of life [22].

Severity of age-related SNHL increases with age, thus the hearing health care requirements of adults are likely to change as they get older. Davis et al [12] showed that approximately 5% of 55-64 year olds experience a significant hearing impairment of ≥35 decibels (dB) in both ears. This increases for those between 65-69 years and between 70-74 years, with the prevalence of a significant hearing loss of ≥35dB hearing level (HL) rising to 15% and 20%, respectively. Adults over the age of 65 years with hearing loss are statistically more likely to benefit from amplification than those younger than 65 years [12]. As such, hearing aid intervention and support related to hearing aids are likely to be appropriate forms of hearing health care for adults in their mid-60s and 70s. However, 1 in 3 adults between 55-64 years experiences hearing losses between 20-34dB [23]. Although classed as “mild” impairment, this can still lead to reduced social interaction, participation, and quality of life [12]. Consequently, it is likely that adults in their 50s and early 60s would benefit from hearing screening and advice regarding hearing loss designed to address the onset of age-related hearing difficulties.

Recent evidence suggests that the Internet may provide a means to facilitate communication in people with hearing loss because it removes the auditory barrier [24] and appeals to those with text-based communication preferences [25]. Nevertheless, research focusing on the association between Internet use and hearing impairment has been confined to an adolescent population to date [24]. It is unknown whether these findings are applicable to the majority of people with age-related hearing loss, especially those who are over the age of 50 years and have mild to moderate losses. Although there is some evidence that PC and Internet use is affected by age, SES, and sex [26,27], there is no published evidence examining whether PC and Internet use are affected by hearing difficulty in older adults. As hearing loss is also associated with age, sex, and SES, it is important to examine the combined effects of all four factors on levels of PC skill and Internet use. This will enable any effects of hearing difficulties to be identified while controlling for any confounding demographic factors.

The primary aim of the present study was to explore the relationships among hearing difficulties and both PC skill and Internet use in an older adult population after accounting for the confounding demographic factors of age, SES, and sex. Should hearing difficulties be related to levels of PC skill and Internet use, it is hypothesized that individuals with hearing difficulty will have greater PC skill and Internet use than those reporting no difficulty. Furthermore, those effects may be greater as the degree of hearing difficulty increases.

Level of PC skill was defined in this study by using a three-category scale (ie, never used a computer, beginner, or competent). The category “never used a computer” is clear. However, the distinction between the “beginner” and the “competent” PC user categories is less well defined. As degree of computer use has been demonstrated to affect computer attitude measures including confidence with computers [28,29], a secondary aim was to verify this three-category PC skill scale by examining user confidence with PCs.

## Methods

### Postal Questionnaire

The primary measure was a 16-item postal questionnaire (Appendix 1) designed as a recruitment tool for a study assessing the benefits of auditory training for adults aged between 50-74 with hearing loss. The questionnaire included five items on hearing difficulties that were used in a hearing screening questionnaire by Davis and colleagues [12]. Previous research using these hearing screening questions found that reports of slight difficulty were associated with hearing losses <35dBHL at 3kHz, whereas reports of moderate difficulty or greater were predictive of hearing loss ≥35dBHL at 3kHz, with sensitivity of 78% [12]. The postal questionnaire also requested details regarding respondents’ PC skill level, Internet use, demographic information, and permission to contact for further research. No details regarding the auditory training study were included in the postal questionnaire, thus eliminating any potential for the auditory training study to influence questionnaire response rates. Instead, those respondents who agreed to be contacted for further research were later contacted to inform them about the study.

Three family practice physician offices in Nottingham, United Kingdom, participated in study recruitment. Postal questionnaires were sent to a total of 3529 patients on the family practice patient registers aged between 50-74, together with an invite letter from the family practice physician (GP in the United Kingdom) and the lead researcher (MAF). Patients were invited to complete and return the questionnaire in an enclosed reply-paid envelope. Non-respondents were not followed up; non-response was assumed to indicate a desire not to participate in the survey.

#### Participants

The response rate to the postal questionnaire was 36.77% (1298/3529), which is comparable with response rates for a recent national postal survey of family practice patients [30]. A total of 63 questionnaires were excluded from the analysis: 18 because the respondents did not complete the PC or Internet questions and 45 because the respondents fell outside of the target age bracket (50-74 years). Data from 1235 respondents were used in further analyses.

Respondents’ ages ranged from 50-74 years with a mean of 62.2 years (SD6.6 years). There were more female respondents (54.49%, 673/1235) than male (45.26%, 559/1235), but 3/1235 (0.25%) of respondents failed to report their sex. SES was determined by using the Index of Multiple Deprivation (IMD) score based on respondents’ postal codes. The IMD is a measure of deprivation by area, with higher IMD scores equating to lower SES, or greater deprivation. In our sample, IMD scores ranged between 3.65 and 78.37 with a mean of 27.87 (SD17.10). Nationally, IMD scores range between 0.99 and 84.22 [31].

### PC Confidence Questionnaire

PC skill was rated in the postal questionnaire on a three-category scale (ie, never used a computer, beginner, or competent). We aimed to verify this PC skill scale by examining whether individuals’ confidence in using a PC differed significantly among these three categories of PC skill.

A second 11-item PC confidence questionnaire (Appendix 2) was developed to obtain more detailed information regarding respondents’ PC use and was administered to a subsample of the postal questionnaire respondents (n=85) who were invited to take part in the auditory training study. Participants completed the questionnaires in the waiting room. The questionnaire comprised of closed-set questions to assess overall confidence using a PC and confidence in using a keyboard, a mouse, and a laptop track pad. Ratings ranged from 0-3: 0 is not confident at all; 1 is I usually need help; 2 is it takes me a while but I can manage; and 3 is confident. Ratings for the four confidence items were summed to form a PC confidence index that ranged from 0-12 points. These data were used to address our secondary aim, which was to verify the three-category PC skill scale used in the primary postal questionnaire. One respondent did not complete the PC confidence questions; therefore, data from 84 respondents were included in subsequent analyses.

#### Participants

Respondents ranged from 50-74 years with a mean age of 63.8 years (SD6.4). A total of 52/84 (62%) respondents were male and 32/84 (38%) were female. IMD scores ranged from 3.84-67.73 with a mean of 25.14 (SD17.45).

### Statistical Analyses

For all analyses, alpha<.05 was considered statistically significant. Initial correlations using Spearman rank correlation (ρ) were conducted to identify associations among PC skill, Internet use, better-ear hearing difficulty (BEHD), and demographic factors.

Main analyses assessed levels of PC skill and Internet use by reported hearing difficulty. Associated factors of age, SES, and sex were included within these analyses. Initially, univariate explorations were used to assess the individual relationship between each factor and PC skill and Internet use. All factors were then pooled within multivariate regression analyses to control for any confounding effects between factors. Through a backward elimination process, factors that were considered to be statistically significant (Wald z-statistic; *P<*.05) were retained within the multivariate logistic regression analyses, and the Akaike information criterion (AIC) was then used to assess relative goodness-of-fit to determine an optimized model. Finally, respondents were divided by median age to investigate the relationship among hearing difficulties and levels of PC skill and Internet use in the “younger” (50-62 years) and the “older” (63-74 years) portions of the sample.

Secondary analyses assessed the three PC skill levels by using respondents’ PC confidence indexes from the subset of respondents who completed the PC confidence questionnaire (n=84). The PC confidence index for each respondent was compared to their selected PC skill category. Analysis of variance (ANOVA) was used to assess any significant differences between categories. These analyses sought to identify whether self-selected PC skill categories were an accurate reflection of the respondents’ confidence in using a PC.

## Results

### Verification of Self-reported PC Skill Categories

Although verification of the self-reported PC skill categories was a secondary aim, the results for the PC confidence questionnaire respondents (n=84) are presented first because they form the basis of the main analyses to follow.

**Figure 1:**
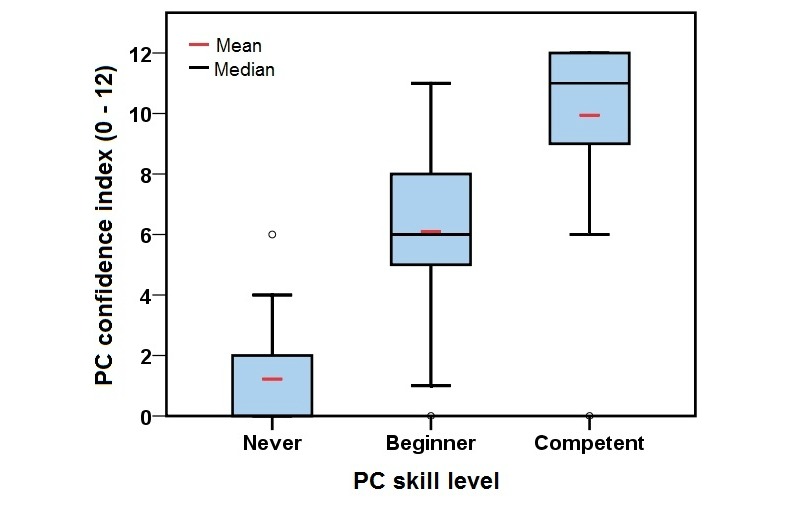
Mean and median PC confidence index by PC skill level for computer confidence questionnaire respondents (n=84).

Mean PC confidence scores by reported PC skill categories are shown in Table 1. A 1-way ANOVA revealed a highly significant between-category difference (*F*_2,81_=69.78; *P<*.001). Respondents who classed themselves as competent PC users scored highest on the PC confidence index, followed by beginners, and then those who had never used a computer (Figure 1). This suggests that respondents were selecting their PC skill levels appropriately based upon their confidence in using a PC and it provides evidence that the PC skill levels selected by respondents in the postal questionnaire were valid reflections of their confidence in using a PC.

### Factors Associated with PC Skill and Internet Use

The primary objective of this study was to explore the effects of BEHD on PC skill and Internet use for the 1235 postal questionnaire respondents.

**Figure 2:**
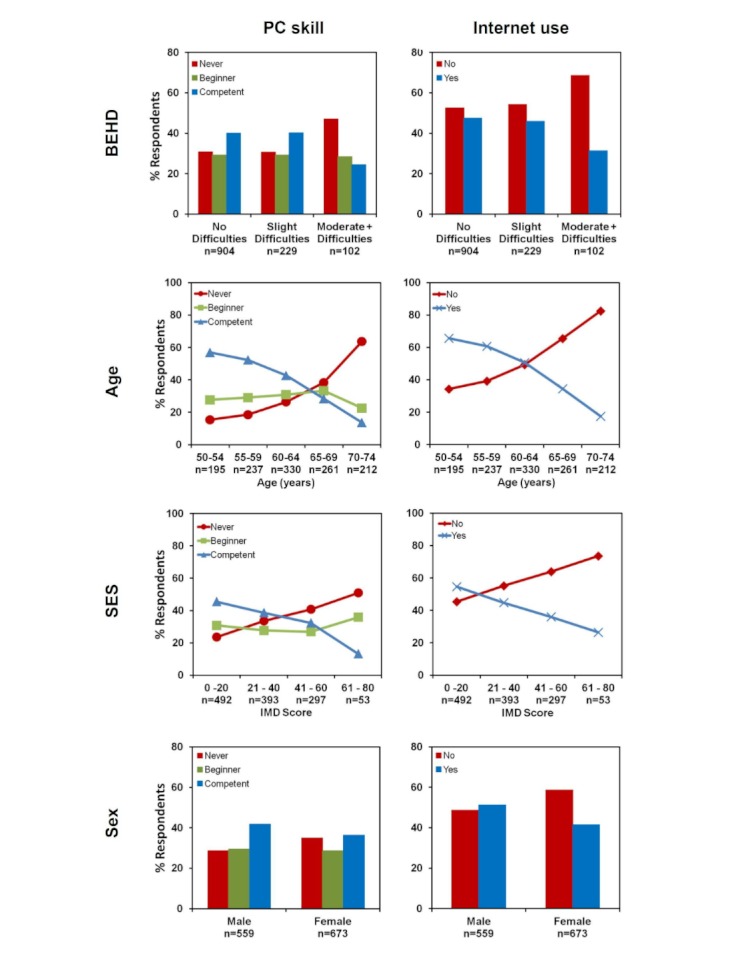
Percentage of postal questionnaire respondents (n = 1235), by PC skill level and Internet use, by better-ear hearing difficulty (BEHD) and demographic factors.

Degree of hearing difficulty was categorised on reported hearing difficulties in the better ear: no hearing difficulty, slight difficulty, and moderate or greater difficulties (moderate+ difficulty). Table 2 shows the frequency of postal questionnaire respondents by BEHD level, age, SES, and sex by PC skill and Internet use. Figure 2 presents these data as percentage of respondents.

Prevalence of hearing difficulties was 26.80% overall (331/1235), which is comparable to data from a large UK hearing population study [12], and was slightly greater in females (27.6%, 186/673) than in males (26.1%, 146/559). For those respondents who reported no hearing difficulty, 45.7% (413/904) were male and 53.9% (487/904) were female. Despite a greater number of female than male respondents overall, for those reporting slight difficulty, 56.4% (128/904) were male and 43.6% (99/904) were female, and for those reporting moderate+ hearing difficulty, 63.4% (64/904) were male and 36.6% (37/904) were female. This represents a statistically significant difference in the prevalence of hearing difficulties, with difficulties being reported more often by male than by female respondents (χ^2^_2_=32.6, *P<*.001).

Over two-thirds of our sample (839/1235, 67.94%) reported being PC users, either beginner or competent, and 45.83% (566/1235) used the Internet. These figures are consistent with existing literature on PC and Internet use in older adults [26,27]. There was a decline in both PC and Internet use with increasing age. In the younger group (50-62 years), PC use (81.0%, 516/637) and Internet use (60.9%, 388/637) was greater than PC use (54.0%, 323/598) and Internet use (29.8%, 178/598) in the older group (63-74 years). For the youngest 5-year age range (50-54 years), 84.6% (165/195) used PCs and 65.6% (128/195) were Internet users, whereas for those respondents in the oldest 5-year age range (70-74 years)—representing the typical ages of first-time hearing aid users—PC use was 36.3% (77/212) and Internet use was 17.5% (37/212).

Respondents who reported slight hearing difficulty were equally likely to rate their PC skill as “never used a computer” (30.6%, 70/229) as those reporting no hearing difficulty (30.8%, 278/904), and equally likely to rate their PC skill as “competent” (40.2%, 92/229) as those reporting no hearing difficulty (40.0%, 362/904). However, those respondents who reported moderate+ hearing difficulty were more likely to rate their PC skill as “never used a computer” (47.1%, 48/102) and less likely to rate their PC skill as “competent” (24.5%, 25/102).

Similarly, for Internet use, respondents who reported slight hearing difficulty were equally likely to use the Internet (45.9%, 105/229) as those reporting no hearing difficulty (47.5%, 429/904). However, those respondents who reported moderate+ hearing difficulty were less likely to report being an Internet user (31.4%, 32/102).

**Table 1:** Mean PC confidence scores (range 0-3; 0=not at all confident to 3=confident) and PC confidence index (range 0-12) by PC skill level for computer confidence questionnaire respondents (n=84).

PC confidence measure	Self-reported PC skills mean (SD)			Mean confidence mean (SD)
	Never	Beginner	Competent	
Keyboard	0.24 (0.56)	2.03 (0.85)	2.75 (0.60)	1.98 (1.17)
Mouse	0.64 (1.01)	2.21 (0.92)	2.91 (0.28)	2.25 (1.09)
Track pad	0.31 (0.79)	1.05 (1.25)	2.19 (0.97)	1.40 (1.28)
Overall PC confidence	0.24 (0.44)	1.23 (0.77)	2.49 (0.66)	1.56 (1.10)
PC confidence index^a^	1.22 (1.93)	6.10 (3.01)	9.94 (2.47)	6.70 (4.21)

^a ^sum of four confidence scores

**Table 2:** Better-ear hearing difficulty (BEHD) and demographics of postal questionnaire respondents (n=1235) by PC skill and Internet use.

Characteristics		Total n (%)	PC skill n (%)			Internet use n (%)	
			Never 396 (32.1)	Beginner 360 (29.1)	Competent 479 (38.8)	No 669 (54.2)	Yes 566 (45.8)
**BEHD**	None	904 (73.20)	278 (70.2)	264 (73.3)	362 (75.6)	475 (71.0)	429 (75.8)
	Slight	229 (18.54)	70 (17.7)	67 (18.6)	92 (19.2)	124 (18.5)	105 (18.6)
	Moderate+	102 (8.26)	48 (12.1)	29 (8.1)	25 (5.2)	70 (10.5)	32 (5.6)
**Age (years)**	50-54	195 (15.79)	30 (7.6)	54 (15.0)	111 (23.2)	67 (10.0)	128 (22.6)
	55-59	237 (19.19)	44 (11.1)	69 (19.2)	124 (25.9)	93 (13.9)	144 (25.5)
	60-64	330 (26.72)	87 (21.9)	102 (28.3)	141 (29.4)	163 (24.4)	167 (29.5)
	65-69	261 (21.11)	100 (25.3)	87 (24.2)	74 (15.4)	171 (25.5)	90 (15.9)
	70-74	212 (17.17)	135 (34.1)	48 (13.3)	29 (6.1)	175 (26.2)	37 (6.5)
**SES**	0-20	492 (39.84)	116 (29.3)	152 (42.2)	224 (46.8)	223 (33.3)	269 (47.5)
	21-40	393 (31.82)	132 (33.3)	109 (30.3)	152 (31.7)	217 (32.4)	176 (31.1)
	41-60	297 (24.05)	121 (30.6)	80 (22.2)	96 (20.0)	190 (28.4)	107 (18.9)
	61-80	53 (4.29)	27 (6.8)	19 (5.3)	7 (1.5)	39 (5.8)	14 (2.5)
**Sex^a^**	Male	559 (45.26)	160 (40.4)	165 (45.8)	234 (48.9)	272 (40.7)	287 (50.7)
	Female	673 (54.49)	235 (59.3)	193 (53.6)	245 (51.1)	394 (58.9)	279 (49.3)

^a ^0.25% (3/1235) of respondents failed to report their sex

### PC Skill Level

Although PC skill was shown to be significantly associated with Internet use in our sample (ρ=0.74, n=1235, *P*<.001), the present research aimed to assess any effects of hearing difficulties on both the skill set and the access of older adults to hearing health care delivered through PCs or the Internet. As such, factors relating to PC skill and Internet use were independently assessed within this study.

Univariate ordinal logistic regression analyses were conducted to examine any significant effects of BEHD, age, SES, and sex on reported PC skill. Results revealed a significant effect of moderate+ hearing difficulty (odds ratio [OR]=0.49, 95% confidence interval [CI] 0.34-0.72, *P*=.001), but no effect of slight hearing difficulty (OR=1.00, 95% CI 0.76-1.30, *P*=.97) on PC skill. There were also significant effects of age (OR=0.90, 95% CI 0.88-0.91, *P*<.001), SES (OR=0.98, 95% CI 0.97-0.99, *P*<.001), and sex (OR=0.77, 95% CI 0.63-0.95, *P*=.015) on PC skill.

All factors were combined within a multivariate ordinal logistic regression model to assess the effects of hearing difficulty on PC skill while controlling for any confounding effects attributable to age, SES, and sex. To aid interpretation, PC skill was categorized as either PC “use” (those with beginner or competent PC skills) or “non-use” (respondents who have never used a computer). Within the categories of BEHD and sex, “none” (no difficulty) and “male” were used as the baselines for comparison, respectively. Results are presented in Table 3 for the whole sample, and for the younger and older groups.

**Table 3:** Logistic regression coefficients (beta) and odds ratio estimates from the multivariate ordinal logistic regression models for factors affecting PC skill level.

Characteristics		All (50-74 years) n=1235			Younger (50-62 years) n=637			Older (63-74 years) n=598		
		beta	Odds ratio (95% CI)	*P*	beta	Odds ratio (95% CI)	*P*	beta	Odds ratio (95% CI)	*P*
**BEHD**	None	–	1.00 (–)	–	–	1.00 (–)	–	–	1.00 (–)	–
	Slight	.13	1.14 (0.86-1.51)	.37	‑.24	0.79 (0.52-1.20)	.26	.45	1.57 (1.06-2.30)	.02
	Moderate+	‑.54	0.58 (0.39-0.87)	.008	‑.74	0.47 (0.26-0.86)	.008	‑.36	0.70 (0.40-1.20)	.20
**Age**		‑.11	0.90 (0.88-0.91)	<.001	‑.04	0.96 (0.92-0.99)	.05	‑.16	0.85 (0.81-0.89)	<.001
**SES**		‑.02	0.98 (0.97-0.99)	<.001	‑.02	0.98 (0.97-0.99)	<.001	‑.02	0.98 (0.97-0.99)	<.001
**Sex**	Male	–	1.00 (–)	–	–	1.00 (–)	–	–	1.00 (–)	–
	Female	‑.28	0.75 (0.61-0.94)	.01	‑.19	0.83 (0.61-1.12)	.23	‑.39	0.68 (0.49-0.93)	.02

#### All Respondents

Results from the multivariate analyses revealed a significant effect of moderate+ hearing difficulty on PC skill level when no difficulty was used as the baseline measure (OR=0.58, 95% CI 0.39-0.87, *P*=.008). This suggests that the odds of being a PC user are 0.58 times less for those reporting moderate+ hearing difficulty than those reporting no hearing difficulty. There was no significant effect of slight hearing difficulty (OR=1.14, 95% CI 0.86-1.51, *P*=.37), suggesting that PC skill did not significantly differ between respondents with slight hearing difficulty and those with no hearing difficulty.

Despite the slight hearing difficulty category showing no significant association with PC skill, a comparison using the likelihood ratio test (LRT) on AIC estimates of a model with and without BEHD revealed a significant difference between models (LRT=8.71, df=2, *P*=.01), suggesting that BEHD was a significant factor associated with PC skill in the multivariate model.

#### Younger Group (50-62 Years)

There was a significant effect of moderate+ hearing difficulty (OR=0.47, 95% CI 0.26-0.86, *P*=.01), but no significant effect of slight hearing difficulty (OR=0.79, 95% CI 0.52-1.20, *P*=.26) on PC skill level in the younger group.

Although age and SES were significant factors related to PC skill, there was no difference between levels of PC skill for males and females (OR=0.83, 95% CI 0.61-1.12, *P*=.23). Further analysis by using the LRT on AIC estimates showed a model including the factor sex did not perform significantly better than a model with this factor removed (LRT=1.47, df=1, *P*=.23). Therefore, sex was eliminated from the final model.

The final model showed a significant effect of moderate+ hearing difficulty (OR=0.49, 95% CI 0.27-0.89; *P*=.02), age (OR=0.96, 95% CI 0.92-0.99; *P*=.04), and SES (OR=0.98, 95% CI 0.97-0.99; *P*<.001) on PC skill level. These results suggest that for those younger respondents with moderate+ hearing difficulty, the odds of being a PC user over a non-user were significantly less than for those with no difficulty hearing.

#### Older Group (63-74 Years)

For the older group, there were no significant effects of moderate+ hearing difficulty (OR=0.70, CI 0.40-1.20, *P*=.20) on PC skill. However, for those with slight hearing difficulty, the odds of being a PC user over a non-user were significantly greater (OR=1.57, 95% CI 1.06-2.30, *P*=.02) than for those with no hearing difficulty.

### Internet Use

Univariate logistic regression analyses revealed a significant effect of moderate+ hearing difficulty (OR=0.51, 95% CI 0.32-0.78, *P*=.01), but no effect of slight hearing difficulty (OR=0.94, 95% CI 0.70-1.25, *P*=.66), on Internet use. Thus, there were no differences between Internet use for respondents with slight hearing difficulty and those with no hearing difficulty. However, for those respondents reporting moderate+ hearing difficulty, the odds of being an Internet user were 0.51 times less than those reporting no hearing difficulty. Demographic factors of age (OR=0.90, 95% CI 0.88-0.91, *P*<.001), SES (OR=0.98, 95% CI 0.97-0.99, *P*<.001), and sex (OR=0.67, 95% CI 0.54-0.84, *P*=.001) were also shown to be significantly associated with Internet use in the univariate analyses.

All factors were combined within a multivariate ordinal logistic regression model to assess the effects of hearing difficulty on Internet use while controlling for any confounding effects of age, SES, and sex. Results are presented in Table 4 for the whole sample, and the younger and older groups.

**Table 4:** Logistic regression coefficients (beta) and odds ratio estimates from the multivariate logistic regression model for factors affecting Internet use.

Characteristics		All (50-74 years) n=1235			Younger (50-62 years) n=637			Older (63-74 years) n=598		
		beta	Odds ratio (95% CI)	*P*	beta	Odds ratio (95% CI)	*P*	beta	Odds ratio (95% CI)	*P*
**BEHD**	None	–	1.00 (–)	–	–	1.00 (–)	–	–	1.00 (–)	–
	Slight	.02	1.02 (0.74-1.14)	.90	‑.35	0.70 (0.45-1.10)	.12	.45	1.57 (0.99-2.47)	.05
	Moderate+	‑.55	0.58 (0.36-0.92)	.02	‑.74	0.32 (0.16-0.62)	.001	.05	1.05 (0.54-1.96)	.88
**Age**		‑.11	0.89 (0.88-0.91)	<.001	‑.04	0.96 (0.92-0.99)	.04	‑.13	0.87 (0.83-0.92)	<.001
**SES**		‑.02	0.98 (0.97-0.98)	<.001	‑.02	0.98 (0.97-0.99)	<.001	‑.03	0.97 (0.96-0.98)	<.001
**Sex**	Male	–	1.00 (–)	–	–	1.00 (–)	–	–	1.00 (–)	–
	Female	‑.47	0.62 (0.49-0.80)	<.001	‑.45	0.64 (0.46-0.89)	.009	‑.51	0.60 (0.41-0.87)	.007

#### All Respondents

There was a significant effect of moderate+ hearing difficulty on Internet use (OR=0.58, 95% CI 0.36-0.92, *P*=.02). However, as with the findings for PC skill, there was no significant effect of slight hearing difficulty (OR=1.02, 95% CI 0.74-1.14, *P*=.90), suggesting Internet use did not significantly differ between those respondents with slight hearing difficulty and those with no hearing difficulty.

Further analysis by using error sum of squares (SSE) on AIC estimates revealed that a model including BEHD did not perform significantly better than a model with this factor removed (SSE=5.49, df=2, *P*=.06). As such, BEHD was eliminated from the final model.

Results from the final model show a significant effect of age (OR=0.89, 95% CI 0.88-0.91, *P*<.001), SES (OR=0.98, 95% CI 0.97-0.98, *P*<.001), and sex (OR=0.64, 95% CI 0.50-0.82, *P*<.001) on Internet use. These results suggest that despite respondents with slight hearing difficulty having significantly greater odds of being Internet users than those with no hearing difficulty overall, this was not a significant predictor of Internet use in the multivariate model.

#### Younger Group (50-62 Years)

There was a significant effect of moderate+ hearing difficulty (OR=0.32, 95% CI 0.16-0.62, *P*=.001), but no significant effect of slight hearing difficulty (OR=0.70, 95% CI 0.45-1.10, *P*=.12) on Internet use. This indicates that for the younger group, the odds of being an Internet user over a non-user were significantly less for respondents with moderate+ hearing difficulties when compared with those with no hearing difficulty. These results are similar to those shown for PC skill.

#### Older Group (63-74 Years)

For the older group, there was a significant effect of slight hearing difficulty (OR=1.57, 95% CI 0.99-2.47, *P*=.05), but no significant effect of moderate+ hearing difficulty (OR=1.05, 95% CI 0.54-1.96, *P*=.89) on Internet use. This indicates that for those reporting slight hearing difficulties in the older group, the odds of being an Internet user over a non-user were greater than for those with no hearing difficulty. These results are similar to those for PC skill.

## Discussion

Hearing-related interventions are of particular relevance to older adults because of the increasing prevalence of hearing loss with age, particularly beyond 50 years. Our postal questionnaire survey identified that for the younger group (aged 50-62 years), levels of PC and Internet use were high (81.0% and 60.9%, respectively), whereas for the older group (63-74 years), PC and Internet use was considerably less (54.0% and 29.8%).

Hearing difficulties were shown to be significantly associated with PC skill and Internet use after controlling for effects of age, sex, and SES, all of which are significant factors in hearing loss. The findings can be categorized into two main effects. First, those with slight hearing difficulties had significantly increased PC and Internet use compared with those with no hearing difficulties in the older group only. Second, those with moderate+ hearing difficulty (ie, moderate/great/cannot hear at all) had lower PC and Internet use than those with no hearing difficulties, both for the whole sample and within the younger group. Although literature exists that examines the effects of age on PC and Internet use [26,27], any interplay between demographic factors and hearing difficulties on PC and Internet use have not been examined previously in an adult population to our knowledge. The current study offers a novel investigation as to how demographic factors and hearing difficulties are associated with PC skill and Internet use in a large sample of adults aged 50-74 years(n=1235). This provides evidence to underpin and establish the potential for PC and Internet delivery of hearing health care information and interventions for people with hearing difficulties.

The postal questionnaire sample was similar to those in other studies for response rate [30], prevalence of hearing difficulties [12], and PC and Internet use [26,27]. For example, a European study of adults aged 55-74 years reported Internet use at 37% [26], which was similar to the equivalent age group in our sample (35.5%). A US study with a similar sample size for those over 50 years (no upper limit), showed PC use (including iPad and smartphone use) was 73% and Internet use was 48.6% [27], whereas Internet use in our present sample (50-74 years) was 45.8%.

PC skill and Internet use differed according to age and hearing difficulty in our sample. Respondents with slight hearing difficulties in the older group had increased odds of PC use (OR=1.57, 95% CI 1.06-2.30, *P*=.02) and Internet use (OR=1.57, 95% CI 0.99-2.47, *P*=.05) compared with those reporting no hearing difficulties. This is consistent with previous research reporting increased motivation to use the Internet and increased Internet use in adolescents with hearing loss [24]. One reason for the increased Internet use suggested by Barak and Sadovsky [24] is that PCs and the Internet provide people with hearing loss with a means of communication that is primarily visual. This puts them on an equal communication basis with people without hearing loss so their hearing disability ceases to be an issue when communicating in the visual modality. The use of PCs and the Internet has been shown to be important for the communication needs of older adults with hearing loss. Pilling and Barrett [25] showed that text-based communication preferences of older adults with severe to profound hearing loss differed from those of adolescents with comparable hearing losses. Almost half (44%) of the adults aged 50-69, preferred to communicate through email, whereas adolescents aged 15-18 preferred short message service (SMS) text messages (64%). Only 12% of adolescents selected email as their preferred means of text-based communication.

There are 10 million people in the United Kingdom with significant hearing loss, yet only 2 million have hearing aids and just 1.4 million use their hearing aids regularly [14]. The result is a huge unmet need, which could—at least in part—be addressed by online hearing health care either outside or within the current UK model of National Health Service (NHS) hearing care provision. Untreated hearing loss can be a significant problem for both the person with hearing loss and their family and friends, leading to reduced social interaction, participation, and quality of life [12]. Given that the typical age for first fitting of hearing aids is approximately 74 years and with many of these adults experiencing hearing difficulties for an average of 10 years prior to hearing aid fitting [12], untreated hearing loss is of particular importance for those aged 63-74 years. Our findings indicate that PC and Internet use was greater in this age group for those with early signs of hearing difficulties. This suggests that PCs and the Internet could be used to target specific hearing health care needs for this group.

Two potential health care approaches for the older group with slight hearing difficulties are delivery of effective information and hearing screening. Information and advice to educate these older adults about the effects of hearing loss and the benefits of hearing aids could be delivered in the form of short, easy-to-use video tutorials [9]. It is anticipated that this increased awareness will encourage at least some in this age group to seek appropriate interventions at an earlier age than is typical, with all the attendant benefits such as improved communication, participation, and quality of life [13]. This may be as simple as understanding the process of how to get a hearing test and what to expect afterwards [32]. Such information could be accessed easily either through a PC or streamed online through the Internet. In addition, levels of user interactivity with health care information can be substantially increased by delivery through PCs and the Internet, with users being able to revisit and review previously encountered material, which may lead to greater learning [33].

Remote hearing screening through the Internet is another highly relevant intervention for this cohort, and it has been shown to provide early benefits [12]. Screening enables the detection of those who are likely to benefit from hearing aid amplification and can help encourage individuals to attend audiology services for appropriate support. There are already good examples of screening initiatives delivered through the Internet and telephone. To date, the hearing check provided by Action on Hearing Loss has provided nearly one million hearing checks [3].

Finally, for those with mild hearing losses where hearing aids are unlikely to offer substantial benefit compared to those with greater levels of hearing loss, PCs and the Internet could be used to deliver alternative interventions such as auditory training to help alleviate the difficulties associated with hearing loss [17,18]. The main advantage of Internet delivery of auditory training is that accessibility to this intervention would be relatively high. A further advantage of online delivery of auditory training, which is not routinely available in the NHS, is that it would not necessarily require the user to access this through audiology services.

In the younger age group (50-62 years), in which PC and Internet use was highest, there was no difference in PC or Internet use between respondents with slight hearing difficulty and those with no hearing difficulty. We suggest that this is a result of there being a high level of PC and Internet use in this group already, which masks specific differences due to hearing difficulties. The relatively high skill set and Internet use in this age group suggests that online delivery of hearing health care is feasible for this younger age group with hearing loss. Appropriate online hearing health care for these adults within this age range may include online information and advice on the detrimental effects of untreated hearing loss, with a view to promote earlier awareness of hearing-related interventions including auditory training.

For respondents in the older age group (63-74 years), in which PC and Internet use was lowest, those with moderate+ hearing difficulties were equally likely to use PCs and the Internet as those with no hearing difficulties. This suggests that online hearing-related interventions may not be particularly effective if targeting this group as a whole. Subsequently, hearing health care tailored for those with moderate+ hearing losses, which is most likely to be hearing aid provision and information or advice relating to amplification, would be best supplemented with additional methods of information support (eg, printed materials or video tutorials delivered through DVD for those who do not or cannot access PC and Internet technology). Of course, for the 30% in the older age group who do use the Internet, information and advice would still be a valid option. The relatively low PC and Internet use in this group does not mean that online delivery of hearing health care will not be suitable for most of this cohort in the future. As PC and Internet use is becoming more prevalent in older adults over time, online delivery of hearing health care may provide a cost-effective, efficient method of providing hearing health care for older adults with a moderate+ hearing difficulties in future years [9].

Some limitations of the present study should be highlighted. First, our sample is limited to a small demographic (adults aged 50-74 years living in Nottingham, United Kingdom) because we were specifically interested in the pre-hearing aid user population. Nevertheless, our respondents have been shown to be representative of published data in terms of the prevalence of hearing loss and PC and Internet use. The World Health Organization lists adult-onset hearing loss as the most common cause of disability worldwide, with presbycusis (age-related hearing loss) the leading cause of adult-onset hearing loss [34]. Because eHealth offers the potential for hearing health care to be delivered globally, further studies may wish to assess the relationships among hearing difficulties and PC and Internet use in geographically remote and hard-to-reach populations, or those under the age of 50 years with significant hearing loss. Second, our postal questionnaire did not request details about our respondent’s employment history. Those employed in manual professions are less likely to have used PCs or to use the Internet regularly at work [35]. This may have affected overall levels of PC skill and Internet use. Future studies may wish to control for employment as a potential confounding factor. Third, PC skill and Internet use were assessed subjectively in this study. Internet use was dichotomized as either “yes” or “no” without any information on frequency or proficiency of Internet use or their information delivery preferences because this was not the purpose of the postal questionnaire at that time. When considering online delivery of hearing health care for older adults, basic levels of PC skill and Internet proficiency are likely to be sufficient to allow access to online information and intervention [17]. However, further investigation of older adults’ information technology skill levels, access, and information delivery preferences will help to inform specific delivery and content of online hearing health care for those older than 50 years.

In the present digital era, delivery of health care information and intervention through PCs and the Internet is common and the traditional method of clinical or medical health care delivery is supplemented increasingly by online information and support [3-10]. Advantages of PC and Internet delivery of supplementary information, hearing screening, and other interventions include the ability to reach those who do not or cannot present to an audiologist. This is of particular relevance given that approximately 47% of adults aged 55-74 years who visit their family practice physician about their hearing difficulties fail to be referred to an audiologist or hearing specialist [12]. Further advantages include both time and cost efficiency, with patients being able to access information at a time or place that suits them. Findings from this study suggest that delivery of hearing health care through the Internet can potentially target a substantial proportion of adults aged 50-74 years with age-related hearing loss, many of whom may not typically present to an audiologist. Therefore, PC and Internet delivery of hearing health care could help address the huge unmet need in those over the age of 50 years who have hearing loss, but do not currently have access to intervention or receive intervention.

## Multimedia Appendix 1

Postal questionnaire (hearing questions).

## Multimedia Appendix 2

Computer confidence questionnaire.

## Abbreviations

AIC: Akaike information criterion
ANOVA: analysis of variance
BEHD: better-ear hearing difficulty
CI: confidence interval
GP: general practitioner
HL: hearing level
IMD: Index of Multiple Deprivation
LRT: likelihood ratio test
NHS: National Health Service
NIHR: National Institute for Health Research
OR: odds ratio
PC: personal computer
SES: socioeconomic status
SMS: short message service (text message for mobile phones)
SNHL: sensorineural hearing loss
SSE: error sum of squares
