# Supplementary material for: Computer Skills and Internet Use in Adults Aged 50-74 Years: Influence of Hearing Difficulties
Source: J Med Internet Res. 2012 Aug 24;14(4):e113. doi: 10.2196/jmir.2036 (PMC3510684; doi:10.2196/jmir.2036)
Supplement: Supplementary file 1 [file jmir_v14i4e113_app1.pdf]

Hearing Questions

If you normally wear a hearing aid, please answer the following questions as if **NOT** wearing a hearing aid. Please put a (✓) in the relevant box along with any further comments.

		(✓)
**Q1.**	**Do you have any difficulty with your hearing?**	
	No	☐
	Yes	☐
**Q2.**	**Do you find it very difficult to follow a conversation if there is background noise (such as TV, radio, children playing)?**	
	No	☐
	Yes	☐
**Q3.**	**How well do you hear someone talking to you when that person is sitting… …on your RIGHT SIDE in a quiet room?**	
	With no difficulty	☐
	With slight difficulty	☐
	With moderate difficulty	☐
	With great difficulty	☐
	Cannot hear at all	☐
**Q4.**	**…on your LEFT SIDE in a quiet room?**	
	With no difficulty	☐
	With slight difficulty	☐
	With moderate difficulty	☐
	With great difficulty	☐
	Cannot hear at all	☐
**Q5.**	**Do you use a hearing aid?**	
	No, never used a hearing aid	☐
	Yes, nowadays	☐
	Yes, in the past (more than 2 years ago)	☐
**Q6.**	**Would you be happy for us to contact you about further research studies?**	
	No	☐
	Yes	☐

Thank you for your help.

Please return the questionnaire in the reply paid envelope. No stamp is needed
