# Supplementary material for: Computer Skills and Internet Use in Adults Aged 50-74 Years: Influence of Hearing Difficulties
Source: J Med Internet Res. 2012 Aug 24;14(4):e113. doi: 10.2196/jmir.2036 (PMC3510684; doi:10.2196/jmir.2036)
Supplement: Supplementary file 2 [file jmir_v14i4e113_app2.pdf]

Assessing your basic computer skills

This questionnaire is intended to assess what you already know regarding basic computers skills. Please, read carefully each question and **tick** the most appropriate answer:

1. How would you rate your computer skill level?
   - Never used a computer O
   - Beginner O
   - Competent O

2. How confident are you with computers?
   - Not confident at all O
   - I usually need help O
   - It depends on the task O
   - Confident O

3. How confident are you using the:

	(a) keyboard	(b) mouse	(c) track pad
Not confident at all	O	O	O
I usually need help	O	O	O
It takes me a while but I can manage	O	O	O
Confident	O	O	O

4. Do you own a computer?
   - Yes O
   - No O

4a. If yes, how often do you use computers?
   - Never O
   - Monthly O
   - Weekly O
   - Daily O

4b. If not, do you have access to any other computer? (e.g., public library)
   - Yes O
   - No O

5. Do you have someone you can ask for help if you have any questions relating to computers?
   - Yes O Who? ____________________________________
   - No O

6. Have you previously used computer-based training packages (e.g. the Nintendo DS Brain Training)?
   - Yes O
   - No O
   - If yes, which packages have you used?

Thank you for filling out this questionnaire
